# The comparison of extraction methods of ganjiang decoction based on fingerprint, quantitative analysis and pharmacodynamics

**DOI:** 10.1186/s13020-020-00355-5

**Published:** 2020-08-05

**Authors:** Yanyan Wei, Ning Jiang, Tuo Liu, Chang Liu, Wen Xiao, Likeng Liang, Tongming Li, Yang Yu

**Affiliations:** 1 School of Pharmaceutical Sciences, Guangzhou University of Chinese Medicine, Guangzhou, Guangdong 510006 China; 2 The Second Affiliated Hospital of Guangzhou, University of Chinese Medicine, Guangzhou, Guangdong 510006 China

**Keywords:** Ganjiang decoction, Fingerprint, Quantitative analysis, Pharmacodynamics

## Abstract

**Background:**

Ulcerative colitis (UC) is a chronic nonspecific inflammatory disease of the colon and rectum with unknown etiology, and its symptoms include bloody diarrhea, abdominal pain, and hematochezia. Traditional Chinese medicine compound has a good therapeutic, multi-target effect on UC. Ganjiang decoction (GD), which is a traditional classic prescription in China, contains Zingiberis Rhizoma, Angelicae Sinensis Radix, Coptidis Rhizoma, Phellodendri Chinensis Cortex, Sanguisorbae Radix, Granati Pericarpium, and Asini Corii Colla and could be used to treat symptoms of UC. This study aimed to conduct a preliminary study before GD colon-targeted preparation, to explore the relationship between extraction method and efficacy of GD.

**Methods:**

High-performance liquid chromatography (HPLC) was used for the fingerprinting of five preparation methods of GD. HPLC and gas chromatography were used to quantitatively analyze the important chemical components of GD and compare their differences. Mice with UC induced by dextran sulphate sodium salt received the extracts from the five preparation methods of GD via gavage. Disease activity index (DAI) score, colonic length, relative weight of spleen, pathological analysis results, inflammatory factors, therapeutic effect of the five preparation methods of GD, and their relationship with extraction process were compared.

**Results:**

Cluster analysis revealed that the content of the components extracted by traditional extraction methods was significantly different from the other four methods. The third and fifth preparation methods extracted Coptidis Rhizoma and Phellodendri Chinensis Cortex with 50% ethanol to obtain more alkaloids. In the fourth and fifth methods, more volatile oils were detected by adding Zingiberis Rhizoma and Angelicae Sinensis Radix fine powder. According to DAI score, colonic length, relative weight of spleen, pathological analysis results, and inflammatory factors, the third method showed a good therapeutic effect, while the fifth method had the best therapeutic effect.

**Conclusions:**

The results showed that the difference of the five extracts of GD in the efficacy of DSS-induced UC in mice was closely related to the extraction method. Our study improved the extraction process of GD and provided a foundation for the process of enteric-soluble preparations and a new idea for traditional Chinese medicine compound preparation. 
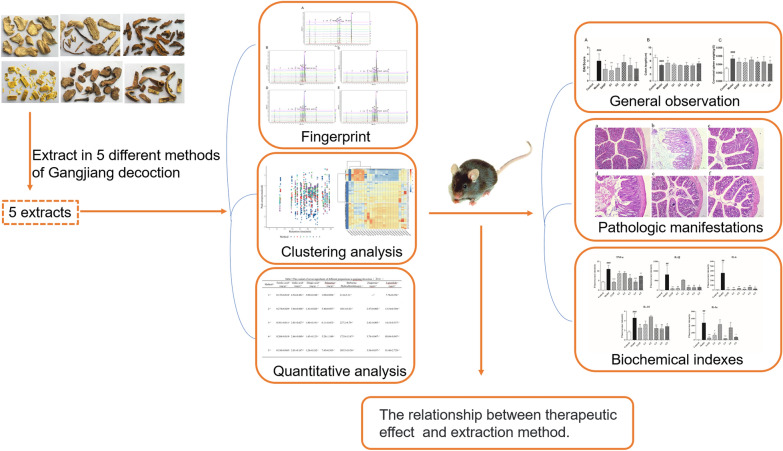

## Background

Ganjiang decoction (GD), which is a Chinese herbal formula, was first recorded in the Tang Dynasty ‘Thousand-Golden-Prescriptions (Beiji Qianjin Yao Fang)’ volume 14, prescription of Zingiberis Rhizoma, Angelicae Sinensis Radix, Coptidis Rhizoma, Phellodendri Chinensis Cortex, Sanguisorbae Radix, Granati Pericarpium, and Asini Corii Colla. Clinically, it was mainly used for the treatment of small intestine distension and pain, hematochezia or loose stools, and intestinal peristalsis, which are clinical manifestations similar to those of ulcerative colitis (UC). The combination of traditional Chinese medicinal materials played different roles in the prescription of GD. Zingiberis Rhizoma and Angelicae Sinensis Radix were used as analgesic and anti-inflammatory agents [1]. Coptidis Rhizoma and Phellodendri Chinensis Cortex showed an anti-inflammatory and bacteriostatic effect [2–6]. Sanguisorbae Radix and Granati Pericarpium had anti-inflammatory and anti-astringent effects  [7, 8].

UC, which is a type of inflammatory bowel disease, is an inflammation of the colon with recurrent attacks and lesions mainly affecting the mucous membranes of the colon and rectum. Currently, the incidence of UC has increased worldwide, with a prevalence of 24.3, 19.2, and 6.3 in 100,000 people in Europe, North America, and Asia, respectively [9, 10]. It has been listed as one of the modern intractable diseases by the World Health Organization.

Although the exact etiology and pathogenesis of UC remain unclear, UC is considered an autoimmune disease, which is affected by multiple factors and closely related to genetic, environmental, immune, and inflammatory mediators; intestinal microecological disorder; intestinal mucosal barrier injury; cytokine imbalance; and other factors [11]. As the mechanism of UC needs to be clarified, appropriate drug therapy remains to be established. Currently, chemical drugs used to treat UC mainly include aminosalicylic acid, glucocorticoids, and immunosuppressants, whose effects are not satisfactory. Moreover, these agents are associated with relapse and more long-term side effects [12, 13].

Traditional Chinese medicine compound contains a variety of ingredients and thus has multi-targeted effect and certain advantages [14–16]. Ancient Chinese medicine used the human body to evaluate the effect of drug use, and oral decoction was primarily administered. However, understanding of the etiology and pathology was limited, and an oral decoction also had its limitations. Moreover, traditional decoction was often extracted by water, and the active ingredients were not dissolved completely, which limited the curative effect. UC occurs in the sigmoid colon and colon; thus, the effect of traditional decoction could not reach the affected area. Hence, traditional Chinese medicine development has always aimed to improve pharmacological, chemical, and targeted drug delivery to increase patients’ treatment compliance.

According to the clinical needs, GD is intended to be developed as a colon-targeted agent. This study aimed to conduct a preliminary study before GD colon-targeted preparation, to explore the relationship between extraction method and efficacy of GD. High-performance liquid chromatography (HPLC) was used for the fingerprinting of five preparation methods of GD, and HPLC and gas chromatography (GC) were used to compare the differences of the chemical components, thereby providing a foundation for the process of enteric-soluble preparations. Moreover, the therapeutic effects of the five preparation methods of GD on experimental colitis mice were compared. This study also provided a new idea for traditional Chinese medicine compound preparation.

## Materials and methods

### Chemicals and reagents

**Fig. 1:**
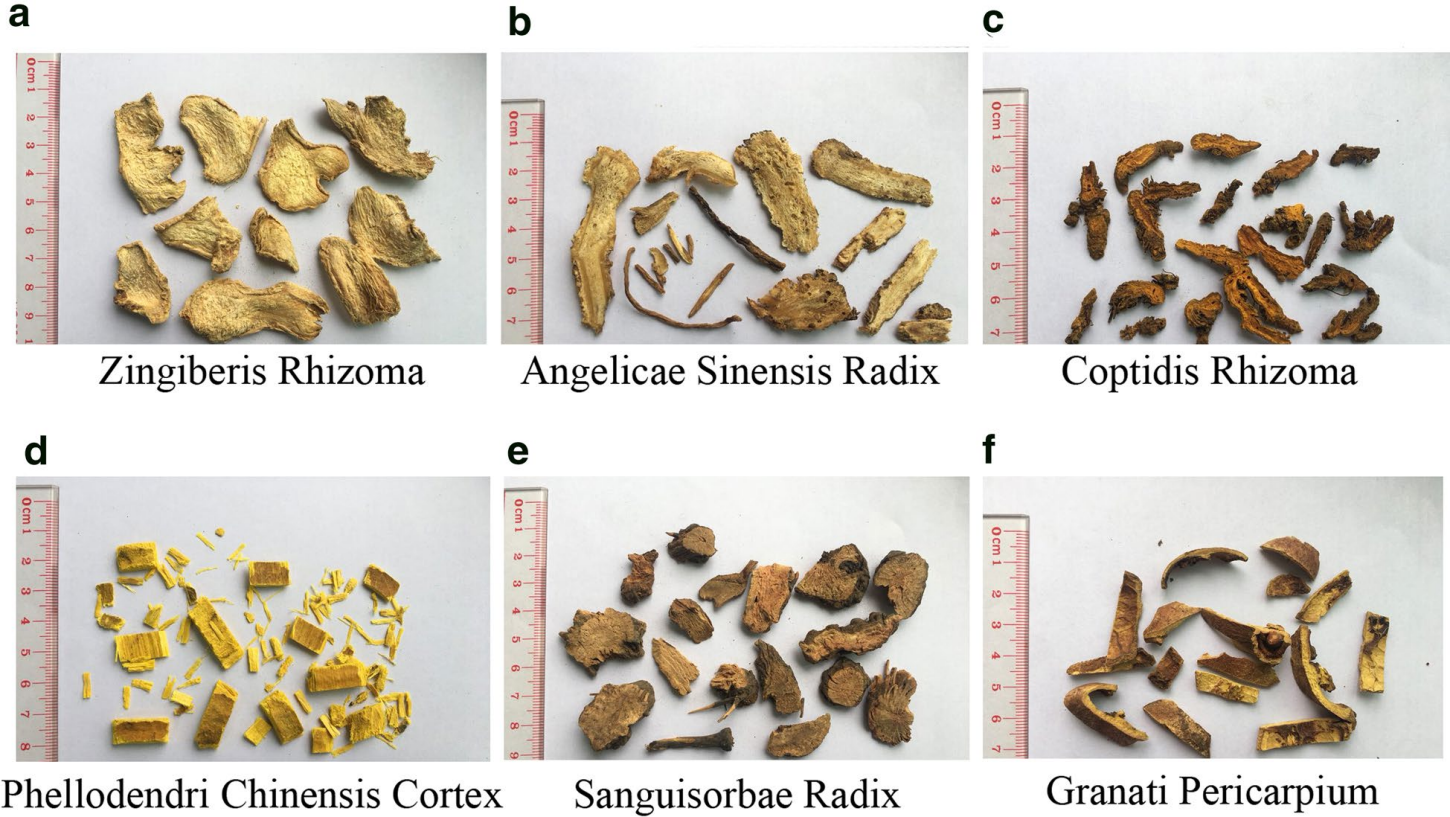
Characteristics of Chinese traditional herbal medicines. Zingiberis Rhizoma (**a**), Angelicae Sinensis Radix (**b**), Coptidis Rhizoma (**c**), Phellodendri Chinensis Cortex (**d**), Sanguisorbae Radix (**e**), and Granati Pericarpium (**f**) were identified by professional and technical personnel

Zingiberis Rhizoma, Angelicae Sinensis Radix, Coptidis Rhizoma, Phellodendri Chinensis Cortex, Sanguisorbae Radix, and Granati Pericarpium were identified by Dr. Peng Guangtian of Guangzhou University of Chinese Medicine (Fig. 1). Zingiberis Rhizoma, Angelicae Sinensis Radix, and Granati Pericarpium were purchased from Anhui Jishun traditional Chinese Medicine Co., Ltd. (Bozhou, China); Coptidis Rhizoma and Sanguisorbae Radix were purchased from Anhui Jucao Traditional Chinese Medicine Co., Ltd. (Bozhou, China); Phellodendri Chinensis Cortex was purchased from Shanxi HongSen Traditional Chinese Medicine Co., Ltd. (Xian, China); Asini Corii Colla was purchased from Shandong Jishui Ejiao Co., Ltd. (Heze, China). Zingiberone, ferulic acid, palmatine, jatrorrhizine, berberine hydrochloride, gallic acid, ellagic acid, zingerone and ligustilide had a purity of 98%, as determined by HPLC or GC, and were provided by Chengdu Weikeqi Biotechnology Co. Ltd. (Chengdu, China). Methanol and acetonitrile were purchased from Merck & Co. Inc. (Darmstadt, Germany). Phosphoric acid was purchased from Aladdin Reagent Co., Ltd. (Shanghai, China). Dextran sulphate sodium salt (DSS) was provided by Yisheng Biotechnology Co., Ltd. (Shanghai, China). Salicylazosulfapyridine (SASP) enteric-coated tablets were provided by Fuda Pharmaceutical Co., Ltd. (Shanghai, China). All other chemicals were of reagent grade.

### Five preparation methods of ganjiang decoction

GD can be divided into four combinations according to the property, flavor, and pharmacologic action of traditional Chinese medicine. Zingiberis Rhizoma and Angelicae Sinensis Radix are the combination 1, for the warm medicine group. Coptidis Rhizoma and Phellodendri Chinensis Cortex are combination 2, which is the cold medicine group. Combination 3 consist of Sanguisorbae Radix and Granati Pericarpium, which is the astringent group. Combination 4, there is Asini Corii Colla, which is the hemostatic group.

According to the property and flavor of these traditional Chinese medicine and the character of the ingredients contained, GD was prepared by five methods. Method 1 was to extract the whole compound prescription with distilled water. Coptidis Rhizoma and Phellodendri Chinensis Cortex, Sanguisorbae Radix and Granati Pericarpium were extracted respectively in methods 2–5 to prevent the reaction between components. Methods 2–5 were also investigated the effects of different solvents on the extraction efficiency of alkaloids. Some fine powder of Zingiberis Rhizoma and Angelicae Sinensis Radix was added in method 4 and method 5 to increase the volatile oil. The specific preparation method of GD was shown in Table 1.

**Table 1:** Different preparation method of ganjiang decoction

Method	Extraction		
	Reflux extracted with distilled water for 30 min twice, decompressed subsequently, concentrated to a thickening paste, dried at 60 °C.	Reflux extracted with 50% alcohol for 60 min, decompressed subsequently, concentrated to a thickening paste, dried at 60 °C.	Fine powder
1	Extract combination ①②③ together		Combination ④
2	Extract combination ①③ together, Extract combination ② separately		Combination ④
3	Extract combination ①③ together	Combination ②	Combination ④
4	Extract combination ①③ together, Extract combination ② separately		A little Combination ①, Combination ④
5	Extract combination ①③ together	Combination ②	A little Combination ①, Combination ④

Combination ①: Zingiberis Rhizoma and Angelicae Sinensis Radix; Combination ②: Coptidis Rhizoma and Phellodendri Chinensis Cortex; Combination ③: Sanguisorbae Radix and Granati Pericarpium. Combination ④: Asini Corii Colla. The total quantity of Combination ① in method 4 and method 5 was the same as that in other methods. The amount of fine powder added was one-sixth of the amount extracted by solvent

### Fingerprint analysis of ganjiang decoction prepared by the five methods

The final sample obtained by the five extraction methods was the mixture of dried extract and dried medicinal powder. Methanol was added to the samples prepared by the five methods and ultrasonic for 30 min. The supernatant solution was obtained by a 0.22 μm microporous filtration membrane.

The fingerprint and quantitative analyses of GD by HPLC were performed by an LC-20AT HPLC System and LabSolutions chromatographic work station (SHIMADZU Cooperation, Japan); Waters-C_18_ column (250 mm × 4.6 mm × 5 μm) (Waters Cooperation, US) was used. The mobile phases of the fingerprint analysis were composed of methanol (A)–0.1% phosphoric acid water (B), with gradient elution (0–5 min, A 10–15%; 5–25 min, A 15–39%; 25–38 min, A 39–45%; 38–40 min, A 45–48%; 40–50 min, A 48–48%; 50–55 min, A 48–60%) at a flow rate of 0.8 mL/min. The column temperature was 30 °C. The detection UV wavelength was 254 nm, and the injection volume was 10 μL.

The data of 10 batches of samples for five extraction method were imported into the similarity evaluation system software of traditional Chinese medicine chromatographic fingerprint (Version 2012.130723). S1 was set as the reference map, the whole spectrum peak was automatically matched, the control map was generated, and the similarity was calculated.

The peak area of each component was standardized, and hierarchical clustering analysis was performed on the standardized data. In the hierarchical cluster analysis, Euclidean method was adopted. Distance was measured for similarity, and the clustering method was complete linkage. The clustering results were shown in heat map and dendrogram; the heat map represented the standardization coefficient of each component in each sample and the tree graph represented the clustering results.

### Quantitative analysis of ganjiang decoction prepared by the five methods

For the quantitative analysis of ferulic acid, gallic acid, and ellagic acid, the chromatographic conditions were the same as those of the HPLC fingerprint analysis.

For the quantitative analysis of palmatine and berberine hydrochloride, the mobile phases were composed of acetonitrile (A)–0.1% phosphoric acid–0.2% triethylamine–water (B), with gradient elution (0–15 min, A 20–30%; 15–35 min, A 30–40%; 35–50 min, A 40–50%) at a flow rate of 0.5 mL/min. The column temperature was 25 °C. The detection UV wavelength was 254 nm, and the injection volume was 10 μL. Sample preparation was the same as that of fingerprint analysis.

Quantitative analysis of zingerone and ligustilide was performed by GC-2010 plus GC System (SHIMADZU Cooperation, Japan) and a Hp-5 column (30 m × 0.25 mm × 0.25 μm). Gas column flow rate was 1.0 mL/min. For the column temperature, the initial temperature was 120 °C, which was maintained for 2 min; subsequently, the temperature was increased to 220 °C at a rate of 12 °C/min, which was maintained for 3 min. Injection port temperature was 250 °C. Detector temperature was 260 °C. Split-flow injection was employed, with a ratio of 20:1 and the injection volume of 1 μL.

Sample preparation of GC: 1.1 g of five samples were taken in 11 mL petroleum ether. Ultrasonic extracted for 30 min. After filtration, petroleum ether was added for extraction again. Two filtrates were combined, concentrated and fixed to 10 ml with petroleum ether. Take the supernatant and pass the 0.22 μm microporous membrane to obtain the sample solution.

### Animals

Male C57BL/6 mice (18–22 g) were provided by the Experimental Animals Center of Guangzhou University of Chinese Medicine. The animals were in specific pathogen-free laboratory (temperature 25 °C, relative humidity 60%) with alternating light and dark for 12 h and had free access to tap water and food. This study was approved by the Animal Ethics Committee of Guangzhou University of Chinese Medicine (Approved No. S2018021).

### Effect of the different preparation methods of ganjiang decoction on DSS-induced UC in mice

The mice entered the acute induction period after 7 days of adaptive feeding. Before the experiment, the mice fasted, but could not control the water for 12 h. The mice were provided 3% DSS solution to drink freely for 7 days to establish the UC model, except those in the control group. After 7 days, the UC mice were divided into the model group (i.g distilled water), SASP group (i.g SASP 0.0038 g/10 g), and another five groups for five extracts of the different methods of GD (i.g 0.1001 g/10 g) (group 1 (G1), group 2 (G2), group 3 (G3), group 4 (G4), and group 5 (G5)). Control group was given the same amount of distilled water. Each group was given gastric gavage once a day for 7 consecutive days.

The gavage drug was prepared according to the “ Five preparation methods of ganjiang decoction”. Extracts of methods 1–5 was concentrated, the corresponding fine powder was added, mixed into suspension and then gavage. During the modeling and administration period, daily dietary water intake, body weight, and disease active index (DAI) scores (fecal: 0 marks for normal, 1 marks for wet sticky, 2 marks for visible perianal fecal, 3 marks for runny bowel) were recorded. Animals were anesthetized with pentobarbital sodium 24 h after the last intragastric administration of GD. Blood samples were obtained from the aorta abdominalis and centrifuged at 3000 rpm for 10 min. Subsequently, the serum was aspirated and stored at −80 °C. The spleen was quickly removed and weighed, and the entire colon was dissociated. The length of the colon was measured, 1 cm of the diseased colon tissue was cut, and the feces were rinsed; thereafter, the cut tissue was fixed in 4% paraformaldehyde and stored at 4 °C. The rest of the colon tissue was frozen in liquid nitrogen and stored at −80 °C for biochemical analysis.

### Histopathological examination and evaluation of colons

The tissues were fixed in 4% paraformaldehyde and dehydrated successively by a series of ethanol solutions. The specimens were permeated and paraffin-embedded; thereafter, the specimens were cut (4 μm) and stained with hematoxylin–eosin. The stained slices were examined by a pathologist using an Olympus CX31 optical microscope (Olympus Corporation, Tokyo, Japan).

Stained tissues were scored according to the histopathological criteria referenced from Binabaj’s study [17]. Histopathological scores were obtained from the sum of the four scores of inflammatory infiltration, crypt loss, mucosal damage and the range of pathological change. The inflammation score was as follows: 0, no inflammatory infiltration; 1, mild inflammatory infiltration; 2, moderate inflammatory infiltration; 3, severe inflammatory infiltration. The crypt loss score was as follows: 0, normal crypt; 1, one-third loss; 2, two-thirds loss; 3, total crypt loss and intact endothelium; 4, total crypt loss and epithelial loss. Mucosal damage score was as follows: 0, intact mucosal; 1, only mucous layer damage; 2, submucosal injury; 3, muscle and serous membrane damage. The range score of pathological changes was as follows: 0, no pathological changed; 1, 25–50% pathological changed; 3, 51–75% pathological changed; 4, 76–100% pathological changed.

### Biochemical analysis

Levels of TNF-a, IL-1β, IL-6, IL-10, and IL-1α in the colon tissue were determined by enzyme-linked immunosorbent assay (ELISA). The Detecting steps assessed using commercially available kits and quantified according to the manufacturers’ guidelines (R&D Systems, LXSAMSM-06).

### Statistical analysis

Statistical analyses were conducted using SPSS 22.0 Statistical Software, and results were treated with GraphPad Prism 5.0. All data were presented as mean ± standard deviation. Comparison between two groups was examined using the least significant difference (LSD) *t* test. One-way analysis of variance (ANOVA) followed by the LSD test was used to compare multiple groups. *P* < 0.05 was considered statistically significant.

## Results

### Fingerprint analysis of the ganjiang decoction extracts by the five methods

The similarity of the fingerprint of the five preparation methods (10 batches) was in the range of 0.990–1.000 (Table 2).

**Table 2:** The similarities of ten batches of samples of five extraction methods for ganjiang decoction

Sample	Similarities				
	Method 1	Method 2	Method 3	Method 4	Method 5
S1	0.995	0.998	0.998	1.000	0.999
S2	0.993	0.994	1.000	1.000	0.999
S3	0.999	0.999	1.000	0.999	1.000
S4	0.996	0.999	1.000	1.000	1.000
S5	0.999	1.000	1.000	1.000	1.000
S6	0.998	0.999	0.999	1.000	1.000
S7	0.999	1.000	1.000	0.998	1.000
S8	0.999	0.993	0.999	1.000	1.000
S9	0.995	0.999	0.999	1.000	1.000
S10	0.990	0.999	1.000	0.999	1.000

**Fig. 2:**
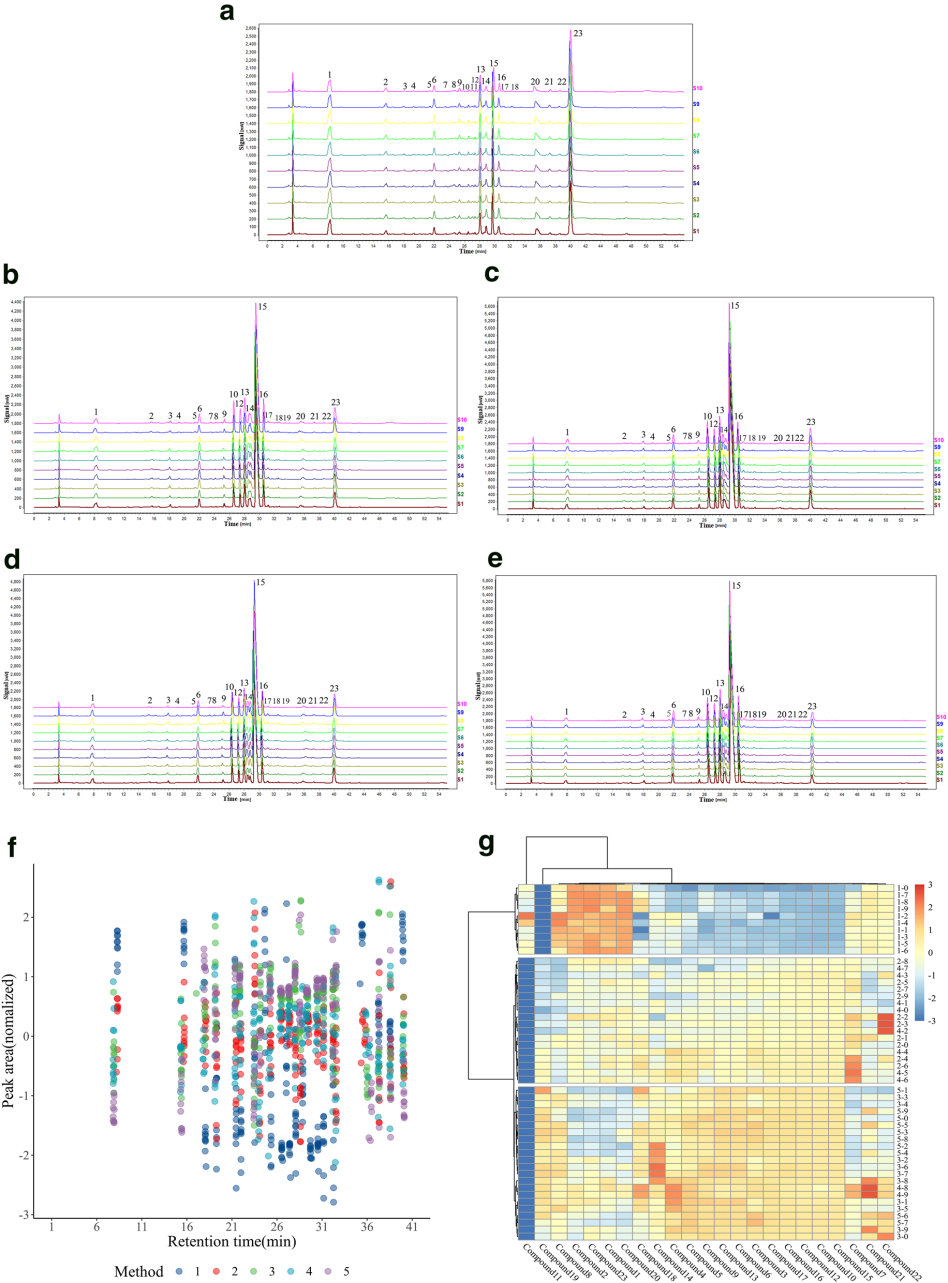
Chromatographic fingerprints and cluster analysis based on the 10 batches of the different extraction of ganjiang decoction. The fingerprint of the extract obtained by the first (**a**), second (**b**), third (**c**), fourth (**d**), and fifth (**e**) extraction method of ganjiang decoction. Scatter diagram was plotted according to the retention time and peak area of common peaks (**f**). Cluster analysis was performed according to the peak area of the common peak, and the height of peak area was expressed by heat map (**g**)

In the first preparation method, 22 common peaks were marked, of which five common peaks were identified: gallic acid (No. 1 peak), jatrorrhizine (No. 14 peak), berberine hydrochloride (No. 15 peak), palmatine (No. 16 peak) and ellagic acid (No. 23 peak) (Fig. 2a).

In the second preparation method, 22 common peaks were marked, of which seven common peaks were identified: gallic acid (No. 1 peak), jatrorrhizine (No. 14 peak), berberine hydrochloride (No. 15 peak), palmatine (No. 16 peak), ferulic acid (No. 18 peak), zingerone (No. 19 peak), and ellagic acid (No. 23 peak) (Fig. 2b).

In the third preparation method, 22 common peaks were marked, of which seven common peaks were identified: gallic acid (No. 1 peak), jatrorrhizine (No. 14 peak), berberine hydrochloride (No. 15 peak), palmatine (No. 16 peak), ferulic acid (No. 18 peak), zingerone (No. 19 peak), and ellagic acid (No. 23 peak) (Fig. 2c).

In the fourth preparation method, 22 common peaks were marked, of which seven common peaks were identified: gallic acid (No. 1 peak), jatrorrhizine (No. 14 peak), berberine hydrochloride (No. 15 peak), palmatine (No. 16 peak), ferulic acid (No. 18 peak), zingerone (No. 19 peak), and ellagic acid (No. 23 peak) (Fig. 2d).

In the fifth preparation method, 22 common peaks were marked, of which seven common peaks were identified: gallic acid (No. 1 peak), jatrorrhizine (No. 14 peak), berberine hydrochloride (No. 15 peak), palmatine (No. 16 peak), ferulic acid (No. 18 peak), zingerone (No. 19 peak), and ellagic acid (No. 23 peak) (Fig. 2e).

Scatter diagrams were drawn according to the peak area and retention time of the samples from the 10 batches of the five extraction methods of GD, and different colors represent the different extraction methods (Fig. 2f). Figure 2g showed the results of cluster analysis. The samples were divided into three clusters based on the tree graph: cluster 1 was mainly the sample of method 1; cluster 2, the samples of methods 2 and 4; and cluster 3, the samples of methods 3 and 5. The analysis indicated that the chemical components extracted by methods 2 and 4 were similar, while those extracted by methods 3 and 5 were similar. Moreover, the heat map also reflected the peak area of each component. The highest contents of compounds 8, 2, 23, 1, and 20 were found in cluster 1, which was followed by cluster 2, and the lowest content was noted in cluster 3. By contrast, compounds 3–6, compounds 9–10, and compounds 12–18 had the highest content in cluster 3, which was followed by cluster 2; the lowest content was found in cluster 1. In addition, compound 11 only appeared in cluster 1, and compound 19 appeared only in clusters 2 and 3.

### Quantitative analysis of ganjiang decoction by HPLC and GC

Based on the aforementioned results of cluster analysis, compound 1 (gallic acid) and compound 23 (ellagic acid) contents were high in method 1 and low in methods 2–5. The contents of compound 15 (berberine hydrochloride), 16 (palmatine), and 18 (ferulic acid) were the highest in methods 3 and 5, which was followed by methods 2 and 4; the lowest was noted in method 1. Thus, HPLC was used for quantitative analysis of these components with large content difference. In methods 4 and 5, a small amount of Zingiberis Rhizoma and Angelicae Sinensis Radix fine powder was added; thus, we used GC to quantitatively analyze the volatile components of zingerone and ligustilide.

**Fig. 3:**
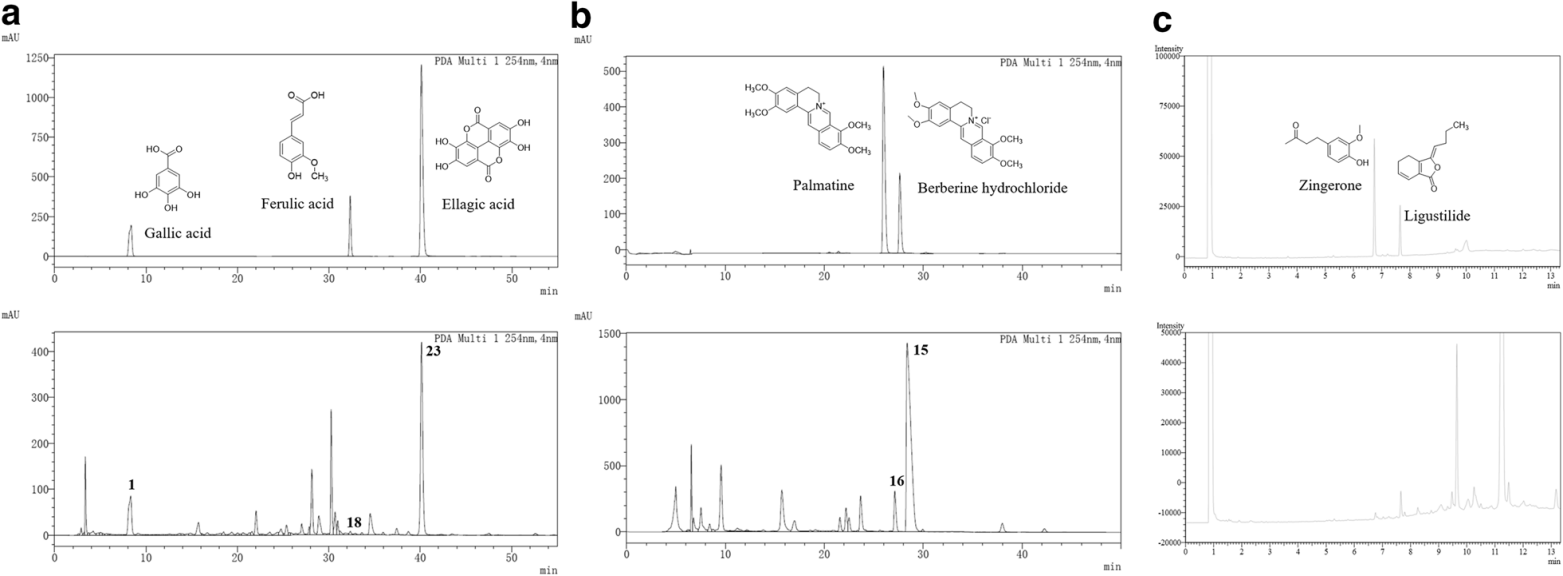
Reference and sample chromatograms in the quantitative analysis. **a** Reference and sample chromatograms of gallic acid, ferulic acid, and ellagic acid in the quantitative analysis by HPLC. **b** Reference and sample chromatograms of palmatine and berberine hydrochloride in the quantitative analysis by HPLC. **c** Reference and sample chromatograms of zingerone and ligustilide in the quantitative analysis by GC. *HPLC* high-performance liquid chromatography, *GC* gas chromatography

The reference chromatogram, calibration curves, and linear range of gallic acid, berberine hydrochloride, palmatine, ferulic acid, ellagic acid, zingerone, and ligustilide were shown in Fig. 3 and Table 3.

**Table 3:** Calibration curves

Standard substance	Linear relation	*R*^*2*^	Liner range (μg/mL)
Ferulic acid	Y = 27206A − 196.23	0.9998	1.5625–100
Gallic acid	Y = 21630A + 39131	0.9999	6.25–200
Ellagic acid	Y = 117421A − 430228	0.9991	6.25–200
Palmatine	Y = 55675A − 70760	0.9994	3.125–200
Berberine Hydrochloride	Y = 13098A − 10110	0.9999	3.125–200
Zingerone	Y = 17424A − 6111.8	0.9984	0.3125–20
Ligustilide	Y = 1437.8A − 1206.1	0.9997	1.5625–100

Y was the corresponding peak area, and A was the concentration

The quantitative results of gallic acid, berberine hydrochloride, palmatine, ferulic acid, ellagic acid, zingerone, and ligustilide in the GD extracts by the five methods were shown in Table 4. In method 1, gallic acid and ellagic acid were the highest content, whereas ferulic acid, palmatine, and berberine hydrochloride were the lowest. In methods 3 and 5, as Coptidis Rhizoma and Phellodendri Chinensis Cortex were extracted separately by 50% alcohol, the alkaloid content, such as palmatine and berberine hydrochloride, was higher. In methods 4 and 5, a small amount of Zingiberis Rhizoma and Angelicae Sinensis Radix powder were added in the extract; thus, the volatile substances, such as zingerone and ligustilide, were higher.

**Table 4:** The content of seven ingredients of different preparations in ganjiang decoction $\left( {\overline{X\,} \, \pm \,S} \right)$

Method	Ferulic acid (mg/g)	Gallic acid (mg/g)	Ellagic acid (mg/g)	Palmatine (mg/g)	Berberine Hydrochloride (mg/g)	Zingerone (μg/g)	Ligustilide (μg/g)
1	0.14 ± 0.01	4.54 ± 0.48	3.09 ± 0.25	1.09 ± 0.09	21.43 ± 3.31	–	7.76 ± 0.26
2	0.27 ± 0.03	3.84 ± 0.07	1.41 ± 0.03	5.46 ± 0.89	168.26 ± 3.63	2.47 ± 0.07	13.10 ± 0.30
3	0.30 ± 0.01	2.83 ± 0.03	1.80 ± 0.19	6.11 ± 0.45	227.19 ± 4.79	2.42 ± 0.01	14.18 ± 0.36
4	0.20 ± 0.02	2.46 ± 0.07	1.45 ± 0.12	5.29 ± 1.10	172.03 ± 13.67	3.76 ± 0.07	88.04 ± 0.95
5	0.34 ± 0.05	2.01 ± 0.17	1.26 ± 0.18	7.43 ± 0.39	305.50 ± 19.58	3.56 ± 0.04	91.46 ± 2.73

mg/g and μg/g refer to the content of components in each gram of dried sample

### The protective effect of ganjiang decoction in mice with DSS-induced UC

Mice were treated with 3%DSS in water for 7 days to induced an acute UC model. Effect of the GD on DSS-induced colitis was evaluated with the DAI score, colon length, corrected spleen weight, and histopathological analysis of colon tissue.

**Fig. 4:**
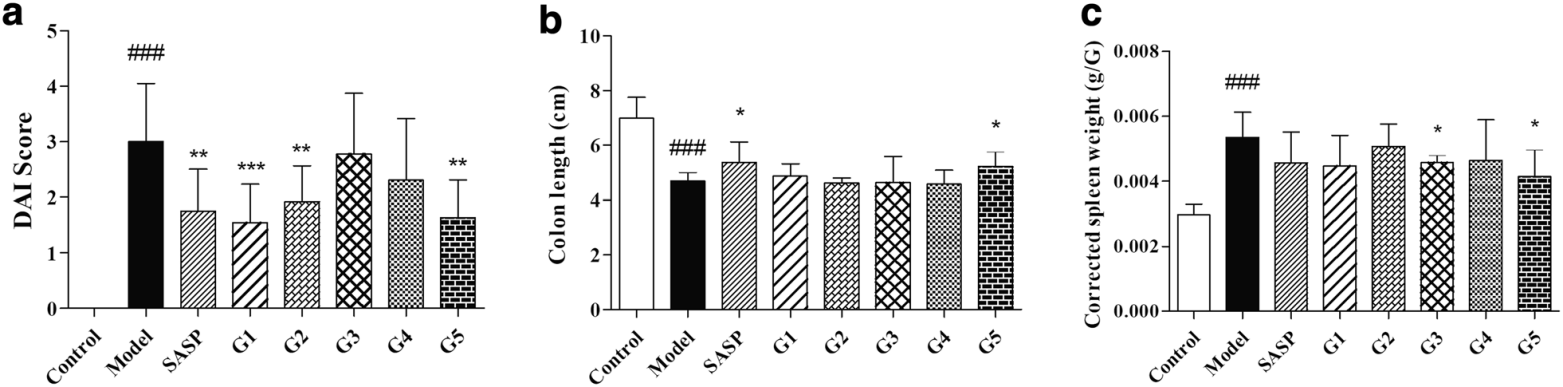
General efficacy of ganjiang decoction in mice with DSS-induced ulcerative colitis. Effect of different extraction methods of ganjiang decoction on DAI score (**a**), colonic length (**b**), and corrected spleen weight (**c**) in mice with ulcerative colitis. Data were presented as *mean ± SD* (n = 6). #*P* < 0.05, ##*P* < 0.01, and ###*P* < 0.001 compared with the control group. *P < 0.05, **P < 0.01, and ***P < 0.001 compared with the model group. *DSS* dextran sulphate sodium salt, *DAI* disease activity index, *SD* standard deviation

All the five extractions of GD reduced the DAI scores of mice with UC, especially the first, second and fifth extracts, where the decline in DAI scores was statistically significant (Fig. 4a). Colon appeared extensive hyperaemia oedema and became shorter in mice with UC. Only the fifth extracts significantly increased the colonic length in mice with UC, compared to the model group (Fig. 4b). All the five methods reduced the corrected spleen weight of mice with UC to different degrees; significant corrected spleen weight was noted in the third and fifth methods (Fig. 4c).

**Fig. 5:**
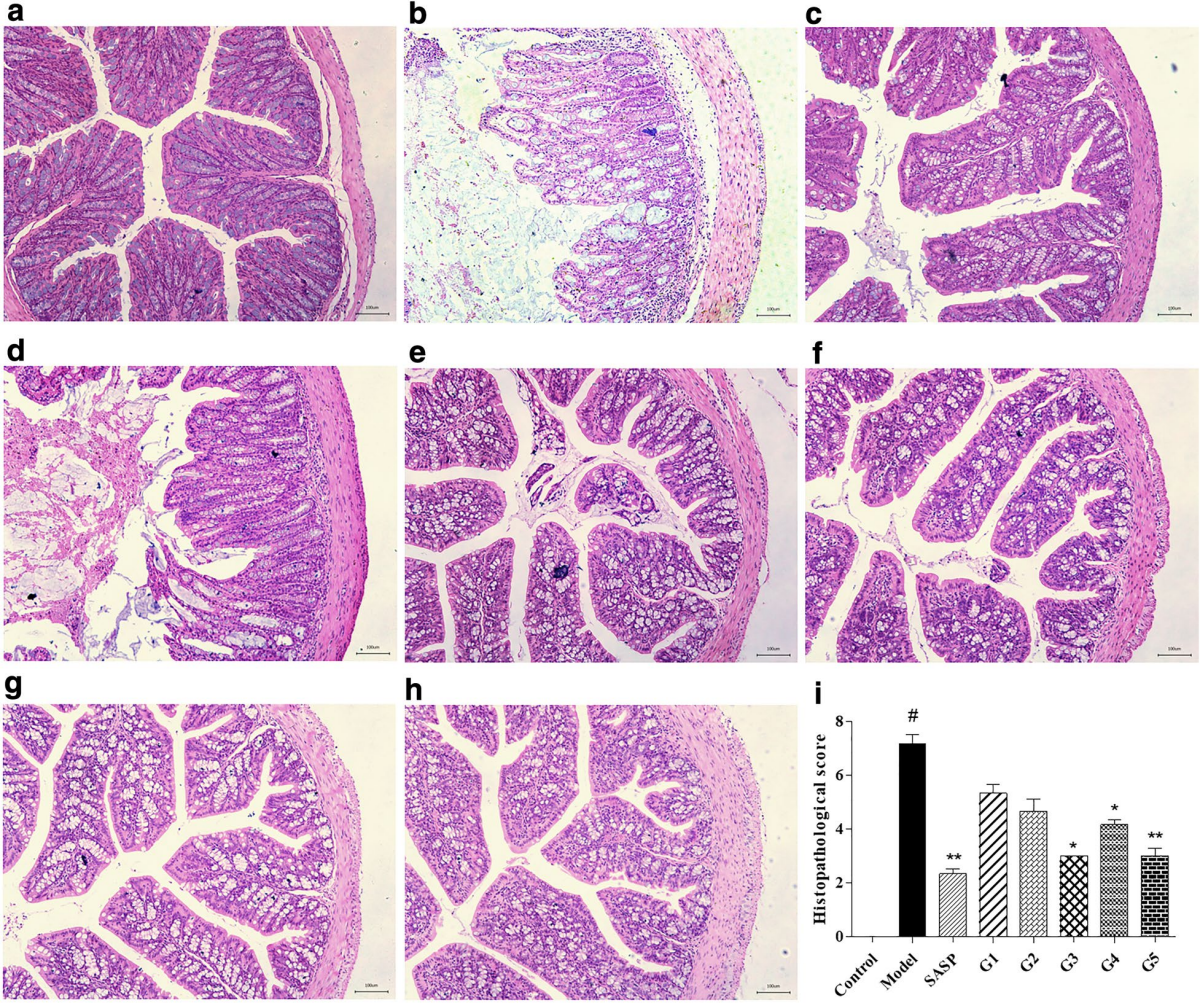
Photomicrographs (× 100, H&E) of the different extraction methods of ganjiang decoction in UC mice. **a** Control group; **b** Model group; **c** SASP group; **d** Group 1; **e** Group 2; **f** Group 3; **g** Group 4; **h** Group 5; **i** Histopathological score. Data were presented as *mean ± SD* (n = 3). #*P* < 0.05, ##*P* < 0.01, and ###*P* < 0.001 compared with the control group. *P < 0.05, **P < 0.01, and ***P < 0.001 compared with the model group. *H&E* hematoxylin and eosin, *UC* ulcerative colitis, *SD* standard deviation. Scale bar: 100 μm

Pathologic manifestations of UC mice in different groups were shown in Fig. 5. Comparing with the control group, there were pathological changes of the colon in the model group, overall damage to the surface epithelium, disruption of cryptal glands, infiltration of inflammatory cells, the loss of goblet cells was observed in the DSS group. In groups 3, 4, and 5, the intestinal epithelial structure of the mucosal layer was complete, and the epithelial cells were normal and arranged tightly. Crypt inflammation was reduced and infiltration of a few lymphocytes was noted.

### Biochemical indexes of mice with DSS-induced UC and the protective effect of ganjiang decoction

**Fig. 6:**
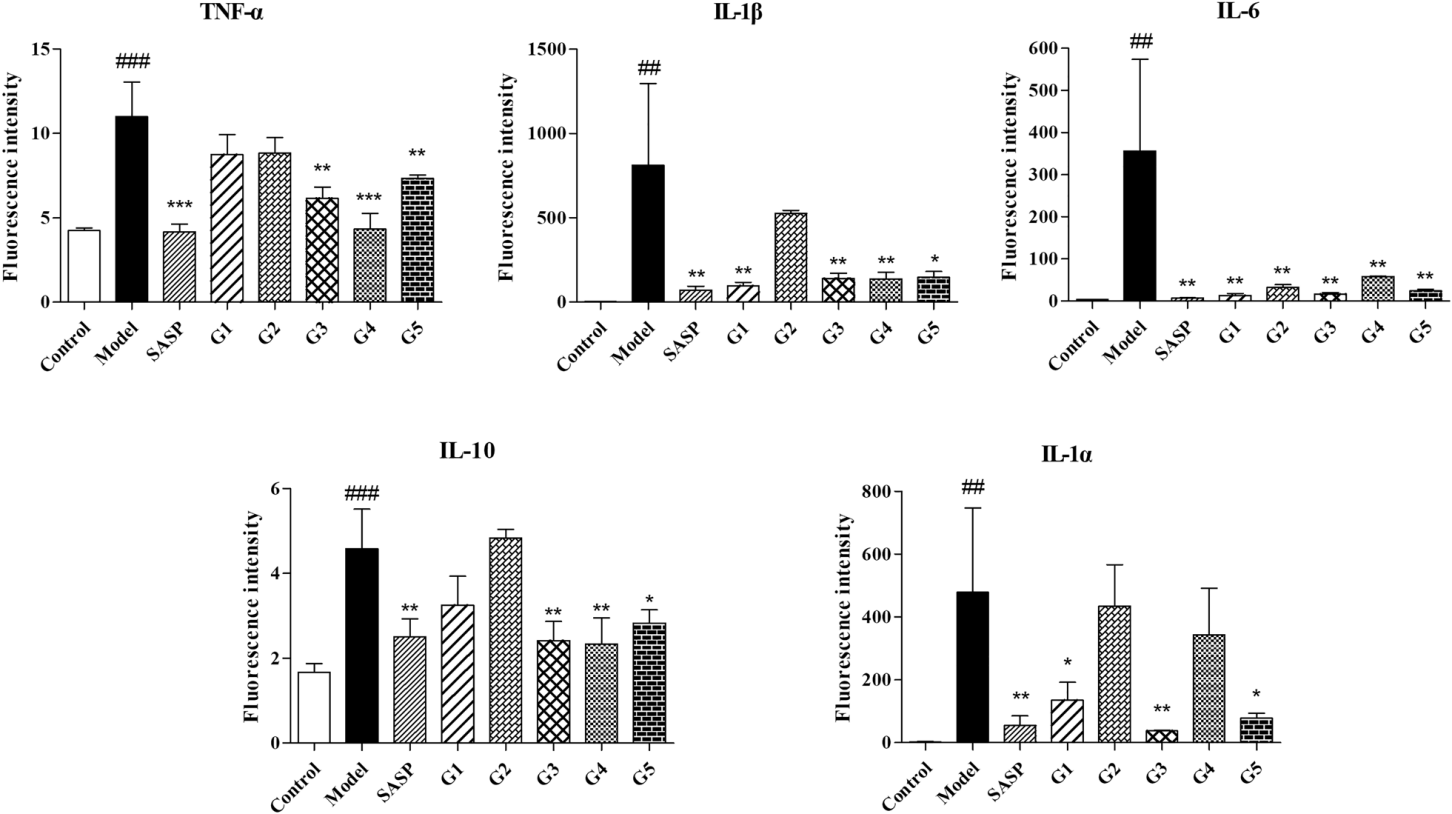
Effects of the five preparation methods of ganjiang decoction on colonic inflammatory factors in UC mice. Data were presented as *mean ± SD* (n = 6). #*P *< 0.05, ##*P *< 0.01, ###*P *< 0.001 compared with the control group. *P < 0.05, ** *P *< 0.01, ***P < 0.001 compared with the model group. *UC* ulcerative colitis, *SD*, standard deviation

The secretion of inflammatory cytokines in mice with ulcerative colitis is disordered, and pro-inflammatory factors increases dramatically. Inflammatory cytokines of mice with DSS-induced UC and the protective effect of GD were shown in Fig. 6.

All the five extractions of GD reduced the levels of TNF-α of mice with UC, especially the third, fourth and fifth extracts, where TNF-α level was reduced significantly; the other methods showed a reduction tendency. The extract of method 1, 3, 4, and 5 could notably reduce the IL-1β level in the colon tissues of the UC mice. All the five extraction methods could significantly reduce the IL-6 level in the colon tissues of the mice. IL-10 levels were significantly decreased in the third, fourth, and fifth groups. The first, third, and fifth extraction methods reduced the IL-1α level in the colon tissues of the mice significantly.

## Discussion

GD was first recorded in the ‘Thousand-Golden-Prescriptions (Beiji Qianjin Yao Fang)’, which was mainly used for the treatment of small intestine distension and pain, hematochezia or loose stools, and intestinal peristalsis, which are clinical manifestations similar to those of UC. Since the onset of UC is mostly in the colon and rectum, which cannot be reached by traditional decoction, we considered to develop GD as a colonic targeted preparation to improve its efficacy. The traditional Chinese medicine decoction contains a variety of ingredients, so it is necessary to make a research on the formulation and selection of ingredients before the preparation development. In this study, pharmacological efficacy was used as the evaluation index to explore and improve the extraction method of GD. Our results showed that the five extraction methods of GD improved the DSS-induced UC in mice to different degrees, and the difference in efficacy was closely related to the extraction methods.

### The increase of alkaloid and volatile oil affected the effect of GD on UV mice

The first extraction method of GD is the most common extraction method of traditional Chinese medicine prescriptions. The cluster and quantitative analyses showed that the content of the chemical components obtained by the first extraction method was significantly different from that obtained by the other four extraction methods. This method did not take into account the relationship between the phytochemical constituents of the Chinese medicinal herbs and the dissolution of active ingredients by an extraction solvent.

Coptidis Rhizoma and Phellodendri Chinensis Cortex contain alkaloids [18, 19], while Granati Pericarpium and Sanguisorbae Radix contain tannins [7, 20]. Alkaloids can react with tannins to form the insoluble precipitate, thus reducing the dissolution efficiency of alkaloids. Hence, in the second, third, fourth, and fifth methods, Coptidis Rhizoma and Phellodendri Chinensis Cortex were extracted by water and 50% ethanol separately, and the alkaloid content was significantly increased. Literature had shown that the extraction efficiency of alkaloids was the highest when the concentration of ethanol was 50%–60% [18]. As 50% ethanol was used as the extraction solvent of Coptidis Rhizoma and Phellodendri Chinensis Cortex, which further improved the extraction efficiency of alkaloids; and the separate extraction of Sanguisorbae Radix and Granati Pericarpium reduced the influence of tannins on alkaloids. Therefore, the palmatine and berberine hydrochloride contents were highest in the third and fifth extraction methods. Studies showed that berberine hydrochloride had beneficial effects on UC, such as suppression of IL-1, IL-1β, IL-6, IL-12, and TNF-α expression [4, 6]. Palmatine also showed an anti-inflammatory action [2, 5]. The high alkaloid content might be the reason why the third and fifth extracts were superior to other extracts in reducing inflammatory factors.

The third and fifth extracts showed the best protective effect against ulcerative colitis based on pathological sections and biochemical indicators. Nevertheless, the fifth method was more advantageous not only considering the DAI score, the length of the colon and the relative weight of the spleen but also because of the greater alkaloids and volatile components in the extract.

In the later stage, we plan to make GD into intestinal soluble pellets. In the optimization of preparation method, the volatile oil of Zingiberis Rhizoma and Angelicae Sinensis Radix was not extracted by reflux method, but some fine powder was added, which could not only improve the content of volatile components, but also reduce the use of excipients in the preparation of pellets. Therefore, the fourth and fifth extraction methods were improved on the basis of the second and third methods. This improvement was to pulverize part of Zingiberis Rhizoma and Angelicae Sinensis Radix into fine powder and directly add into the extraction solution, retaining volatile components such as zingerone and ligustilide. Moreover, the quantitative analysis revealed that zingerone and ligustilide were detected in the second, third, fourth, and fifth extraction methods, and the highest contents were found in the fourth and fifth extraction methods after the addition of Zingiberis Rhizoma and Angelicae Sinensis Radix fine powder. The fifth extraction method had the highest ferulic acid and ligustilide contents and higher zingerone. Ferulic acid showed a good antioxidant effect [21, 22], and ligustilide played a positive role in lipopolysaccharide-induced inflammation in RAW 264.7 macrophages and attenuated vascular inflammation and in activating the defense system of endothelial cells [23, 24]. Moreover, ligustilide showed a good analgesic activity, which inhibited the activation of JNK/c-Jun in spinal cord caused by complete freudian adjuvant (CFA) [25], reduced the inflammatory pain of spinal astrocytes after CFA peripheral injection [26], suppressed microglia cells to mediate inflammatory pain [27]. Other studies reported that ferulic acid and ligustilide could achieve the therapeutic effect on cold-induced vasospasm through the combined action of TRPM8 and TPRA1 [28], which might also be a mechanism for the analgesic effect of the fifth extract. Thus, the increase in these components could explain the better integrated therapeutic effect of the fifth extract on mice with UC.

### Chinese herbal compound prescription has become a new trend in the treatment of UC

SASP has a good therapeutic effect on UC, but there are many reports of adverse reactions, the most common adverse reactions are anal stimulation and abdominal pain [29]. Chinese herbal compound prescription has become a new trend in the treatment of UC because of its advantages of multi-component and multi-target with little side effect. Gegenqinlian decoction could maintain colonic mucosal homeostasis in acute/chronic ulcerative colitis via Notch signal pathway by bidirectional regulation [30]. Sanhuang shu’ai decoction alleviated DSS-induced ulcerative colitis by regulating intestinal microbial flora, inflammatory mediators and cytokines [31]. Studies had shown that many traditional Chinese medicine compounds and formulations could act on NF-κ B p65 directly or indirectly to improve the symptoms of UC [32]. For example, huangqin decoction inhibited the anti-inflammatory effect of RAS-PI3K-AKT-HIF-1α and NF-κB pathway on the UC mice by regulating the intestinal microflora [33]. Huanglian jiedu decoction could alleviate the damage of intestinal mucosal and protect the UC mice by downregulating JAK2 and STAT3 expression via JAK2/STAT3 pathway [34]. In this paper, the extraction method of GD had been improved. The extract of the fifth method contained a variety of active ingredients, which could play a role in multiple phenotypes of UC such as inflammation and abdominal pain, showing the advantages of multi-target of traditional Chinese medicine compound prescription.

## Conclusions

This study showed an improved extraction process of GD based on the fingerprint cluster analysis and content determination and on the therapeutic effect on UC in mice. The results showed that the difference of the five extracts of GD in the efficacy of DSS-induced UC in mice was closely related to the extraction method. These results provided a basis for the enteric preparation of GD and a new idea for traditional Chinese medicine compound preparation.

## Data Availability

Not applicable.

## Abbreviations

UC: Ulcerative colitis
GD: Ganjiang decoction
HPLC: High-performance liquid chromatography
DSS: Dextran sulphate sodium salt
GC: Gas chromatography
SASP: Salicylazosulfapyridine
i.g: Intragastric administration
DAI: Disease active index
H&E: Hematoxylin–eosin
ELISA: Enzyme-linked immunosorbent assay
LSD: Least significant difference
ANOVA: One-way analysis of variance
TNF-α: Tumor necrosis factor α
IL-1β: Interleukin 1β
IL-6: Interleukin 6
IL-10: Interleukin 10
IL-1α: Interleukin 1α
